# Assessing the instantaneous social dilemma on social distancing attitudes and vaccine behavior in disease control

**DOI:** 10.1038/s41598-024-64143-z

**Published:** 2024-06-20

**Authors:** K. M. Ariful Kabir, Jun Tanimoto

**Affiliations:** 1 Department of Mathematics, Bangladesh University of Engineering and Technology, Dhaka, 1000 Bangladesh; 2 Interdisciplinary Graduate School of Engineering Sciences, Kyushu University, Kasuga-Koen, Kasuga-Shi, Fukuoka, 816-8580 Japan; 3 Faculty of Engineering Sciences, Kyushu University, Kasuga-Koen, Kasuga-Shi, Fukuoka, 816-8580 Japan

**Keywords:** Vaccination game, Social distancing, Social dilemma, Pairwise game, Computational biology and bioinformatics, Mathematics and computing

## Abstract

In the face of infectious disease outbreaks, the collective behavior of a society can has a profound impact on the course of the epidemic. This study investigates the instantaneous social dilemma presented by individuals' attitudes toward vaccine behavior and its influence on social distancing as a critical component in disease control strategies. The research employs a multifaceted approach, combining modeling techniques and simulation to comprehensively assess the dynamics between social distancing attitudes and vaccine uptake during disease outbreaks. With respect to modeling, we introduce a new vaccination game (VG) where, unlike conventional VG models, a 2-player and 2-strategy payoff structure is aptly embedded in the individual behavior dynamics. Individuals' willingness to adhere to social distancing measures, such as mask-wearing and physical distancing, is strongly associated with their inclination to receive vaccines. The study reveals that a positive attitude towards social distancing tends to align with a higher likelihood of vaccine acceptance, ultimately contributing to more effective disease control. As the COVID-19 pandemic has demonstrated, swift and coordinated public health measures are essential to curbing the spread of infectious diseases. This study underscores the urgency of addressing the instantaneous social dilemma posed by individuals' attitudes. By understanding the intricate relationship between these factors, policymakers, and healthcare professionals can develop tailored strategies to promote both social distancing compliance and vaccine acceptance, thereby enhancing our ability to control and mitigate the impact of disease outbreaks in the future.

## Introduction

Epidemiological models^1^ are invaluable tools for managing epidemics, providing predictive insights into disease behavior within a population^2^. Epidemiological behavioral models enable health agencies to prepare and implement strategies to reduce infection peaks and their overall impact^3–7^. Existing models offer critical insights into the disease's trajectory during an epidemic outbreak, facilitating rapid and effective decision-making^8^. However, once the immediate epidemiological crisis subsides, the focus shifts towards enhancing these models by integrating new data and insights. This ongoing development aims to create more robust tools to address future epidemics effectively. In this context, we introduce an epidemiological model that addresses two key aspects. Firstly, it considers the introduction of a vaccine during an ongoing epidemic, enabling us to assess the vaccine's impact on disease dynamics^9–12^. Secondly, it considers the influence of cooperative behavior in maintaining social distancing practices among individuals^13–15^.

When an epidemic spreads, governments implement various countermeasures such as mask-wearing, social distancing, lockdowns, hand hygiene, and vaccination^16–19^. Among these measures, vaccines are a cornerstone of pharmaceutical control policies. They not only protect vaccinated individuals from infectious diseases but may also carry some side effects. Rational individuals assess the benefits and adjust their strategies based on the circumstances^20^. However, this self-centered approach can jeopardize public health and hinder the transition to an endemic phase of infectious diseases. For instance, the ongoing waves of COVID-19 infections driven by mutations undermine the progress achieved through effective vaccines, social restrictions, testing, and quarantine measures^21–26^. Vaccination significantly reduces the risk of infection among people. Yet, individuals with egoistic tendencies may opt not to get vaccinated, relying instead on herd immunity generated by others' vaccinations to avoid vaccine side effects^27^. Unfortunately, reducing the vaccination rate among egoistic individuals creates a social dilemma^28,29^. This situation fails to decrease the risk of infection and leads to a substantial increase in infection rates. Those seeking effective responses to infectious diseases should consider alternative non-pharmaceutical control strategies.

In this study, we aimed to gain insight into how individuals make vaccination decisions in the context of perceived vaccine and disease risks. We examined the game theory payoff within the Susceptible-Infected-Recovered-Susceptible (SIRS) model. In the realm of studying both epidemic dynamics and individual vaccination behavior, there exists a substantial body of prior research known as the "vaccination game" (VG), which includes works such as^9–11,19^. What distinguishes our approach from these previous studies is that our VG model effectively incorporates what is referred to as a "2-player & 2-strategy" framework, which specifically allows us to predict the evolving nature of social dilemmas over time. Additionally, we developed an innovative model to analyze the dynamics of social restriction behavior. Our aim was to bridge the gap between dynamic vaccination game models and the pairwise game-theoretic perspective. This approach primarily hinges on integrating a vaccination game model, categorized as either a Donor-Recipient game or a specific Trivial game class.

Our utilization of game theory, grounded in deterministic algorithms, presents a robust framework for comprehending the strategic decision-making processes involved in mitigating disease transmission rates, particularly concerning the practice of social distancing. Game theory furnishes valuable insights into the consequences of modifying multiple parameters, thereby elucidating their pivotal role in understanding the intricacies of infectious disease dynamics. In parallel, we employ a dynamic vaccination game theory rooted in behavioral analysis, wherein individuals' rational choices wield a significant influence over their willingness to engage in vaccination efforts. The nexus between these two models is rooted in their mutually reinforcing attributes. While the pairwise game theory model affords a strategic vantage point for scrutinizing decision-making, the behavioral dynamics model captures the multifaceted and unpredictable nature of real-world vaccine choices, which subsequently impact the dynamics of infections. Our analytical approach focuses on discerning strategic inflection points by tracking dynamic shifts in individuals' attitudes concerning the utility of vaccination in safeguarding their well-being. The amalgamation of these two models facilitates a more comprehensive understanding of the intricate interplay between individual behavior, strategic decision-making, and the dynamics inherent to infectious diseases.

We introduce the intervention epidemiological model that stands apart from existing models such as Fu's^30,31^ and Bauch's^6,10^ by addressing dilemmas at every time step, unlike these models that mainly represent dilemmas in steady-state situations. In conventional vaccination game models, including Fu's^31,32^, the evolutionary game aspect was not explicitly incorporated, leading to an inherent social dilemma structure. Through the concept of Social Efficiency Deficit (SED), we quantified the gap between expected payoffs at Nash equilibrium and those at the Social Optimal case, identifying social dilemmas in the overall episode. However, SED could not pinpoint dilemmas at specific moments within episodes characterized by initial epidemic growth, infection surges, peak times, decline, and disease eradication. Madeo et al.^33^ recently investigated the intricate dynamics between epidemic spread and cooperative behavior using a game-theoretic framework. Crucial insights into the effectiveness of control measures in mitigating the impact of pandemics like COVID-19 are uncovered by integrating evolutionary game theory with epidemic modeling. High performance is demonstrated by the developed model in fitting real measurements of infected, recovered, and deceased individuals throughout the COVID-19 epidemic spread from March 2020 to September 2021 in Italy. By inspiring form^33^, our innovative framework consider $G$, assessing instantaneous dilemma strength that enables us to evaluate the dynamic evolution of social dilemmas explicitly, shedding light on the interplay between epidemics and human behavior. "$G$" is like a score that tells us how strong the dilemma is at a particular time during the epidemic to decide whether to cooperate or not during an epidemic, like following safety measures or getting vaccinated. Thus, "$G$" helps us understand how serious the situation is during an epidemic and whether our actions are making a difference in controlling the spread of the disease. This approach reveals whether a social dilemma is present at any given moment, providing transparency to the dual dynamics of disease and human behavior. In statistical physics, epidemic dynamics are analyzed using compartmental models such as the SIR model, focusing on disease spread within populations. Additionally, evolutionary game theory contributes to this field by examining how behaviors related to disease prevention evolve, highlighting the dynamics of cooperation and defection within populations^16,34,35^.

## Model description

(a) Epidemic model

In this framework, we operate on the premise that individuals can acquire temporary immunity through both vaccination and the natural contraction of the illness. We introduce an SVIRS epidemic model to examine the impact of voluntary vaccination on a localized timescale, where disease transmission and vaccination strategies coexist. Our mean field approximation model delves into the dynamics of imperfect vaccination in a well-mixed, infinite population, adopting a non-demographic perspective. The population is divided into distinct compartments, encompassing susceptible 

**Figure 1:**
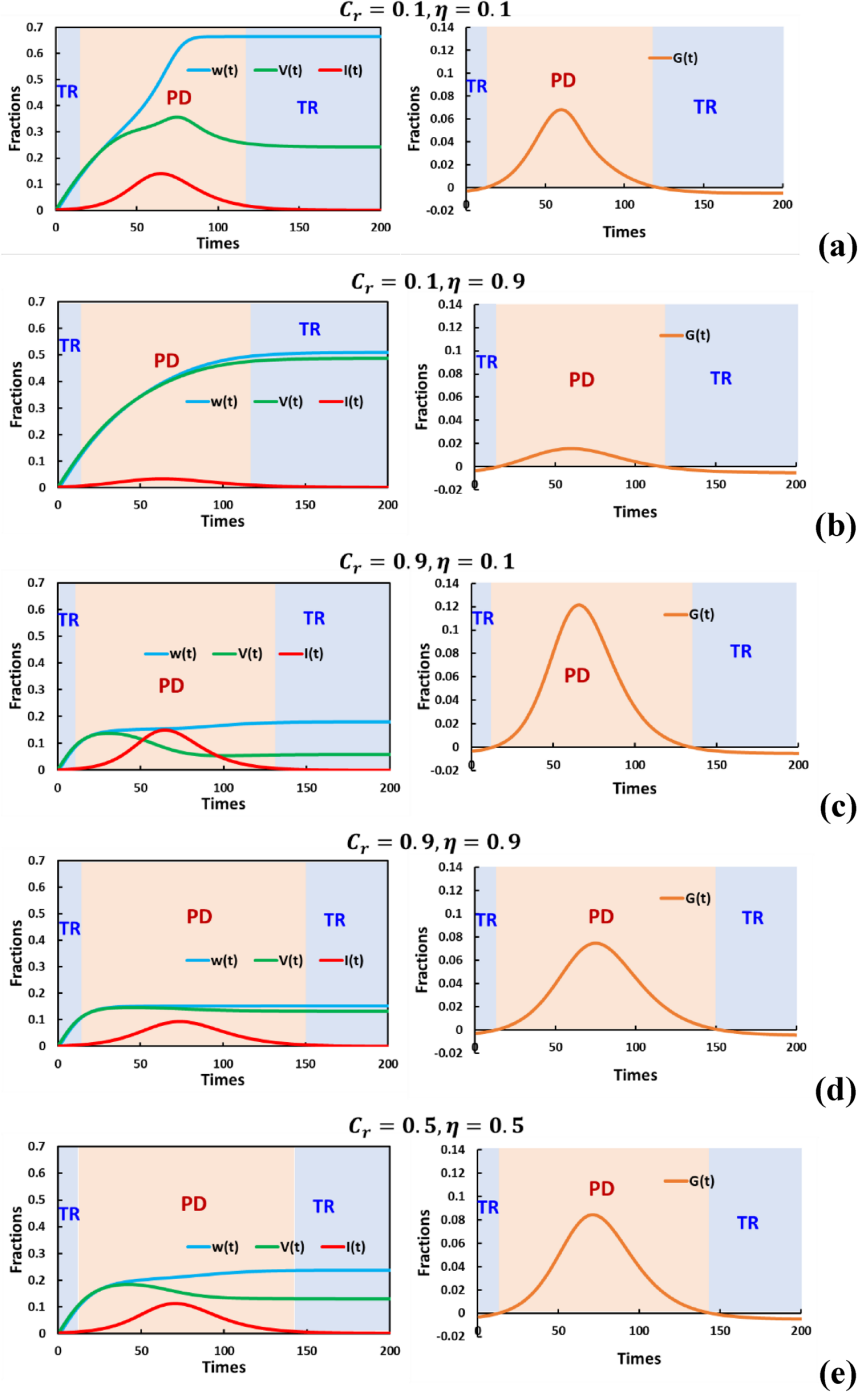
Examine how varying vaccine cost and effectiveness impact disease incidence over time. We're doing this in the context of two different strategies: behavioral vaccination games and social distancing dyadic games, specifically for the case when there is no waning immunity $(p=0.0)$. The aim is to understand how different combinations of vaccine cost and effectiveness influence the dynamics of the disease. In panels (**a**–**e**), we display five specific scenarios: (**a**) $({C}_{r},\eta ) = (\text{0.1,0.1})$: This scenario involves a low-cost vaccine with low efficiency. (**b**) $({C}_{r},\eta ) = (\text{0.1,0.9})$: Here, we have a low-cost vaccine with high efficiency. (**c**) $({C}_{r},\eta ) = (\text{0.9,0.1})$: In this case, the vaccine is expensive but not very effective. (**d**) $({C}_{r},\eta ) = (\text{0.9,0.9})$: This scenario includes a high-cost vaccine with high efficiency. (**e**) $({C}_{r},\eta ) = (\text{0.5,0.5})$: Here, the vaccine has a moderate cost and moderate efficiency. We analyze the impact of varying vaccine cost and efficacy on disease dynamics through behavioral vaccination and social distancing games ($p=0.0$, no waning immunity). Increasing vaccine cost extends the period of the prisoner's dilemma (PD), which is notably seen in panels (**c**), (**d**), and (**e**). While efficiency less affects PD duration, higher $\eta$ reduces infections and epidemic spread frequency. Although $\eta$ might not drastically alter PD duration, it likely affects its severity, indicated by $G(t)$ as a Social Efficiency Deficit (SED). Further evidence is needed for confirmation. Understanding these dynamics is crucial for evaluating social distancing and vaccination strategies in disease control. Others parameters are $\beta =0.8333, \gamma =0.3333, a=0.01, {D}_{g}={D}_{r}=0.5, h=1.0$ and $e=1.0$.

$(S)$, infected $(I)$, recovered $(R)$, and vaccinated $(V)$ individuals. Notably, we define $\eta$ as a constant parameter denoting vaccine efficiency (Table 2). Within the infected category, we further subdivided it into two compartments. The first compartment, ${I}^{S}$, comprises susceptible individuals whom others have infected in the susceptible group. On the other hand, ${I}^{V}$ represents a subgroup of vaccinated individuals who have contracted the illness despite being vaccinated due to vaccine inefficiency. The inter-compartmental relationships we aim to elucidate are visually represented in the schematic diagram in Fig. 1. These relationships are formalized through a set of differential equations.

1 $$\dot{S}=-\beta \left(x\right)S\left({I}^{S}+{I}^{V}\right)+p{R}^{S}+p{R}^{V}-yS,$$

2 $$\dot{V}=-\left(1-\eta \right)\beta \left(x\right)V\left({I}^{S}+{I}^{V}\right)+yS,$$

3 $${\dot{I}}^{S}=\beta \left(x\right)S\left({I}^{S}+{I}^{V}\right)-\gamma {I}^{S},$$

4 $${\dot{I}}^{V}=\left(1-\eta \right)\beta \left(x\right)V\left({I}^{S}+{I}^{V}\right)-\gamma {I}^{V},$$

5 $${\dot{R}}^{S}=\gamma {I}^{S}-p{R}^{S},$$

6 $${\dot{R}}^{V}=\gamma {I}^{V}-p{R}^{V}.$$

In this context, $\beta (x)$ represents the rate at which the disease is transmitted (expressed as occurrences per person per day), $y$ denotes the vaccination rate, and $\gamma$ signifies the rate at which individuals recover from the infectious state. It's important to note that individuals who have acquired immunity, whether through vaccination or recovery from the illness, can become susceptible once more over time due to a gradual decline in their immunity, characterized by the rate parameter $p$.

(b) Behavioral epidemic dynamics (Individual’s Behavior Influences the Epidemic)

Now, let's introduce a game-based framework to model the disease transmission rate, denoted as $\beta (x)$^33^. We assume that an individual's level of cooperation, represented by the variable $x$, reflects their adherence to non-pharmaceutical interventions imposed by public health authorities, such as practices related to social distancing. In general, the greater the compliance of individuals with these behavioral measures, the lower the transmission rate of a disease tends to be. Consequently, we aim to explore how the selection of specific strategies within this game can influence the dynamics of epidemiology. To establish this connection between the game theory and the epidemiological framework, we will formulate an infection rate that depends on the chosen game strategy, as outlined below,

7 $$\beta \left(x\right)={\beta }_{0}\left(1-ex\right); 0\le e\le 1.$$

Here, ${\beta }_{0}$ represents the constant disease transmission rate for a particular disease, and $e$ denotes the proportionality constant of the cooperator. If we consider that the impact of vaccination is stronger than that of social distancing, we can assume that $1-ex\left(t\right)>1-\eta$. We will now proceed by considering a scenario where the population can make rational choices regarding their actions during an epidemic outbreak. Within this context, individuals have the option to manifest two distinct behavioral tendencies, which we will categorize as "cooperation" and "defection". Cooperative behavior encompasses actions that substantially contribute to curbing disease transmission, such as following social distancing guidelines and using personal protective equipment. Building on the notion of cooperative conduct, we introduce a pairwise 2-strategy game into the framework.

8 $$A=\left(\begin{array}{cc}1& -{D}_{r}\\ 1+{D}_{g}& 0\end{array}\right).$$

By rescaling the game's characteristics based on the universal dilemma strength (SD), we can represent the game using two parameters known as "$${D}_{g}$$" and "$${D}_{r}$$". These parameters are referred to as the gamble-intending dilemma and risk-averting dilemma, respectively^36,37^. Depending on the magnitudes of "$${D}_{g}$$" and "$${D}_{r}$$", we can categorize the game into one of four classes: (i)Trivial $$({D}_{g}<0$$ and $${D}_{r}<0)$$—No significant dilemma, (ii) Prisoner's dilemma $$({D}_{g}>0$$ and $${D}_{r}>0)$$—Cooperation is challenging, leading to a dilemma, (iii) Chicken $$({D}_{g}>0$$ and $${D}_{r}<0)$$—A dilemma where one party is risk-averse while the other is not and (iv) $$({D}_{g}<0$$ and $${D}_{r}>0)$$—A dilemma involving coordination between participants. This classification is based on the values of "$${D}_{g}$$" and "$${D}_{r}$$" and characterizes the nature of the game being studied (Table 2).

When the proportion of cooperators is represented as "$x$", and defectors make up the remaining fraction of "$1-x$", the anticipated payoffs (fitness) for both cooperators and defectors, denoted as ${\pi }_{C}$ and ${\pi }_{D}$ respectively, are as follows,

9 $${\pi }_{C}=x-\left(1-x\right){D}_{r},$$

10 $${\pi }_{D}=x\left(1+{D}_{r}\right).$$

The expected payoff ($\overline{\pi }$) (the average social payoff) are formulated as follows,

11 $$\overline{\pi }=x{\pi }_{C}+\left(1-x\right){\pi }_{D}.$$

Utilizing the replicator dynamics^38^ with a foundation in Mean Field Approximation enables us to illustrate the evolutionary dynamics concerning the variable "$x$",

12 $$\dot{x}=x\left({\pi }_{C}-\overline{\pi }\right).$$

Let’s assume that data related to the epidemic can influence the choice of a certain behavior or cooperation strategy. Let, $a$ be real number such that $0<a<1$ and let $w\left(t\right)=V\left(t\right)+{I}^{V}\left(t\right)+{R}^{V}(t)$ and $I\left(t\right)={I}^{S}(t)+{I}^{V}(t)$ be the total fraction of the vaccinated and infected population at time $t.$ We define the functions,

13 $$G\left(t\right)=\left(1-hw(t)\right)I\left(t\right)-a.$$

Here, we employed $a$ as one of the model parameters, which indirectly controls the sign of $G\left(t\right)$, i.e., the social dilemma class, whether Trivial or PD is working behind the disease outbreak. $G(t)$ is a metric that assesses the strength of a social dilemma in the context of an epidemic. It considers the proportion of vaccinated individuals, represented by "$w(t)$" , and the number of infected people, represented by "$I(t)$". By calculating the difference between these factors and subtracting another parameter, "$a$", "G" provides insights into the dynamic evolution of the dilemma, indicating whether the epidemic situation leans towards being manageable or critical.

As previously discussed, the variable "$x$" signifies the proportion of cooperation within a population. A higher degree of cooperation $(0\ll x<1)$ corresponds to a reduced transmission rate, indicating that individuals are more inclined toward "social distancing". Taking this into consideration, we can now explore the relationship between the sign of "$G(t)$" and the specific category of games defined by the payoff matrix "$A$". When we set "${D}_{r}$" equal to "${D}_{g}$", meaning the game class falls into either a Donor and Recipient game with "${D}_{r}={D}_{g}>0$" or a particular Trivial game with "${D}_{r}={D}_{g}<0$", we can make predictions about how the game category evolves during an epidemiological episode as follows (Table 1),

**Table 1:** The game classification evolves during an epidemiological episode.

Total vaccinated $w\left(t\right)$	Infected $I\left(t\right)$	Game structure	What time-stage in an epidemic episode?
[Small]	[Small]	$G\left(t\right)<0$; Trivial	Beginning: less infected and less vaccinated
[Small]	[Large]	$G\left(t\right)\gg 0$; Strong PD	An outbreak starts: Still less people are not vaccinated
[Large]	[Small]	$G\left(t\right)\ll 0$; Strong Trivial	When individuals prioritize vaccination, it can help suppress the spread of diseases. Increased awareness prompts people to adhere to social distancing measures
[Large]	[Large]	$G\left(t\right)>0$; PD	Despite the presence of few vaccinator individuals, outbreaks continue to occur, resulting in a typical social dilemma

In this context, the parameter '$h$' falls within the range of $h\in [\text{0,1}]$ and measures how information regarding the overall proportion of vaccinated individuals affects the population's prioritization of the total count of infected individuals. For instance, when '$h$' approaches 1 and the value of '$w(t)$' is relatively high, the significance of the current infection count '$I(t)$' diminishes. This is particularly noticeable in cases where the disease symptoms among vaccinated individuals are less severe, leading to reduced apprehension. Conversely, with '$h$' nearing zero, the total portion of vaccinated individuals holds no sway over individuals' level of concern regarding the total number of infections.

Now, let,

14 $${D}_{r}={D}_{r}^{0}G\left(t\right),$$

15 $${D}_{g}={D}_{g}^{0}G\left(t\right).$$

Here, ${D}_{r}$ and ${D}_{g}$ are not time-constant, which depend on the functions defined the above equations and, they depend on the epidemiological scenario.

(b)(b) Behavioral vaccination dynamics.

Within the realm of behavioral dynamics^10,11,29–32^, each participant has the autonomy to decide whether or not to engage in the vaccination program. This choice depends on their economic circumstances and the prevailing disease incidence, which can change over time. To participate in the vaccination program, individuals incur a certain cost, denoted as "${C}_{v}$". To facilitate analysis, we've rescaled the relative vaccination cost as "${C}_{r}$", where ${C}_{r}={C}_{v}/{C}_{i}$, with ${C}_{i}$ representing the infection cost, and it is standardized at ${C}_{i}=1.0$. If individuals who are potentially susceptible to the disease decide to participate in the vaccination program at a rate denoted as "$y$", the following equation characterizes the underlying human behavioral process^10,32^,

16 $$\dot{y}=my\left(1-y\right)\left[-{C}_{r}V+I\right].$$

The expression $[-{C}_{r}V + I]$ represents the disparity between the product of the relative vaccination cost $({C}_{r})$ and the number of vaccinated individuals, and the total count of infected individuals ($I\left(t\right)={I}^{S}(t)+{I}^{V}(t)$) at a specific time point denoted as "$t$". In this context, "$m$" serves as a balancing constant, translating the proportion of individuals into the likelihood of switching strategies based on the expected gain in their payoff (Table 2). It's important to note that, for the duration of our analysis, we have assumed a fixed value for "$m$" which is set at 0.2.

**Table 2:** Parameters used in this work with descriptions.

Symbols	Descriptions
${\beta }_{0}$	Constant disease transmission rate
$\beta (x)$	The disease transmission rate accounted for social distancing which represents the rate at which the disease is transmitted
$p$	Waning immunity rate
$y$	Vaccination rate
$\gamma$	Recovery rate from the infectious state
$\eta$	Vaccine efficiency
$x$	Individual's level of cooperation adherence to non-pharmaceutical interventions, such as practices related to social distancing
$e$	Proportionality constant of the cooperator
${D}_{g}$	Gamble-intending dilemma
${D}_{r}$	Risk-averting dilemma
${\pi }_{C}$	Expected payoff for cooperators
${\pi }_{D}$	Expected payoff for defectors
$\overline{\pi }$	Average social payoff
$w\left(t\right)$	fraction of the vaccinated at time $t$
$a$	Control parameter to control the sign of $G\left(t\right)$
$h$	Proportionality constant for vaccinated
${C}_{i}$	Infection cost
${C}_{v}$	Vaccine cost
${C}_{r}$	Relative vaccine cost, ${{C}_{r}={C}_{v}/C}_{i},({C}_{i}=1.0)$
$m$	Balancing constant, translating the proportion of individuals into the rate

## Results and discussion

In this study, we have introduced an epidemiological model that considers individuals' natural or induced immunization. Models incorporating population immunization are significant in assisting public policy efforts to prevent and manage diseases. The ability to forecast disease spread in a population with ongoing immunization processes can greatly aid in planning and selecting optimal disease control strategies. Our proposed model is versatile and applicable to recurring diseases with existing immunization measures and novel and unforeseen diseases. It considers acquired immunity from prior infection and the possibility of developing immunization measures during a disease outbreak, as observed in the case of COVID-19. Further, our model accounts for the non-linear behaviors exhibited by individuals during an epidemic. We have employed game theory to model this behavior, utilizing a dyadic game involving two strategies to represent cooperation and non-cooperation behaviors within the population during an epidemic. By integrating this dyadic game for social distancing strategies and behavioral dynamics into the vaccination game model with the epidemiological framework, we could describe and analyze the impact of social behaviors on disease transmission. Our study elucidates how changes in infection rates correspond to prevailing social behaviors and underscores the role of available epidemiological information in influencing behavioral choices.

### Time series analysis

#### Without waning immunity

In Fig. 1, we are investigating the impact of varying vaccine cost and efficiency on the incidence of a disease over time within the context of two different approaches: behavioral vaccination games and social distancing dyadic games for $p=0.0$ (without waning immunity). The goal is to understand how different combinations of vaccine cost and efficiency influence disease dynamics. In panels (a) to (e) of Fig. 1, we explore five specific scenarios: (a) $({C}_{r}, \eta ) = (0.1, 0.1)$: This scenario represents a situation where the vaccine has low efficiency and is relatively cheap, (b) $({C}_{r}, \eta ) = (0.1, 0.9)$: Here, we have a low-cost vaccine with high efficiency, (c) $({C}_{r}, \eta ) = (0.9, 0.1)$: In this case, the vaccine is expensive but not very effective, (d) $({C}_{r}, \eta ) = (0.9, 0.9)$: This scenario involves a high-cost vaccine with high efficiency and (e) $({C}_{r}, \eta ) = (0.5, 0.5)$: Here, the vaccine has moderate cost and moderate efficiency. In panel (c), where the vaccine is expensive (${C}_{r}=0.9$) and has low efficiency ($\eta =0.1$), we observe a significant peak in infection rates. This suggests that when the vaccine is costly and not very effective, fewer individuals choose to participate in vaccination programs. Consequently, there is a widespread spread of the disease due to the limited protection offered by the vaccine. On the contrary, in panel (b), where the vaccine is low-cost (${C}_{r}=0.1$) and highly effective ($\eta =0.9$), a majority of individuals opt for vaccination. As a result, the disease incidence is substantially reduced. This illustrates that when the vaccine is affordable and provides strong protection, more people are willing to get vaccinated, leading to better control of the disease. These findings highlight the critical interplay between vaccine cost and efficiency in shaping disease dynamics. Lowering vaccine costs and increasing their efficiency can lead to higher vaccination rates and better disease control, while higher costs and lower efficiency can result in more widespread infections.

Let's delve into the analysis of the social distancing game derived from disease incidence and vaccination data over time. When we increase the vaccine cost (${C}_{r}$), it's reasonable to expect that the duration of the period under Prisoner's dilemma (PD) becomes longer. This trend is evident in panels (c), (d), and (e) of our data. Compared to the sensitivity related to vaccine cost, the increase in vaccine efficiency has a lesser impact on extending the time under PD. However, boosting $\eta$ can effectively mitigate the upsurge of infections (I(t)), resulting in a reduction in both the peak infection value and the overall Frequency of Epidemic Spreading. This observation implies that while a higher $\eta$ might not significantly alter the duration of the PD period (i.e., the time when the population faces a dilemma between adopting PD or continuing with normal behavior), it can influence the extent of this dilemma. It's essential to note that we need concrete proof to make a definitive claim about the impact of ETA on the dilemma extent. However, if we consider $G(t)$ as an indicator representing the extent of the dilemma, akin to a Social Efficiency Deficit (SED), our earlier hypothesis seems plausible. In other words, even though a larger Eta may not drastically change the duration of the PD period, it might affect the severity of the dilemma itself. However, this interpretation requires further empirical evidence for confirmation. Understanding these dynamics is vital for assessing the efficiency of strategies related to social distancing and vaccination in managing disease outbreaks.

#### With waning immunity

**Figure 2:**
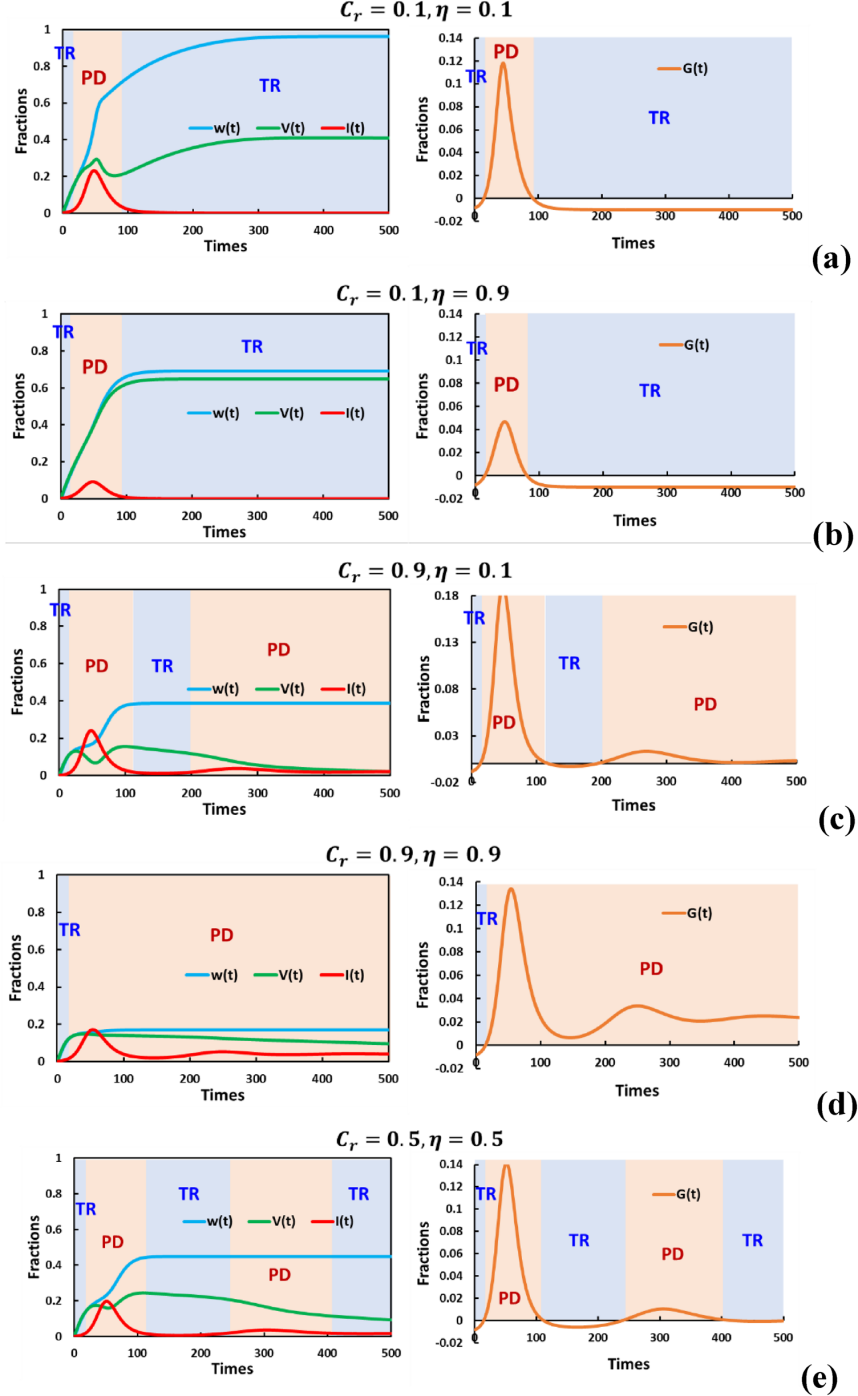
Examine how varying vaccine cost and effectiveness impact disease incidence over time. We're doing this in the context of two different strategies: behavioral vaccination games and social distancing dyadic games, specifically for the case when the waning immunity is $p=0.01$. The aim is to understand how different combinations of vaccine cost and effectiveness influence the dynamics of the disease. In panels (**a**) to (**e**), we display five specific scenarios: (**a**) $({C}_{r},\eta ) = (\text{0.1,0.1})$: This scenario involves a low-cost vaccine with low efficiency. (**b**) $({C}_{r},\eta ) = (\text{0.1,0.9})$: Here, we have a low-cost vaccine with high efficiency. (**c**) $({C}_{r},\eta ) = (\text{0.9,0.1})$: In this case, the vaccine is expensive but not very effective. (**d**) $({C}_{r},\eta ) = (\text{0.9,0.9})$: This scenario includes a high-cost vaccine with high efficiency. (**e**) $({C}_{r},\eta ) = (\text{0.5,0.5})$: Here, the vaccine has a moderate cost and moderate efficiency. Immunity waning ($p=0.01$) significantly alters the dynamics of the Vaccination Game (VG) dilemma structure, mainly when vaccine cost is high (${C}_{r}=0.9$). Hesitancy arises due to perceived cost–benefit parity, causing fluctuations in vaccination commitment ($V(t))$ and multiple periods of Prisoner's Dilemma (PD). This interaction underscores the complex, evolving patterns of social distancing and vaccination compliance in disease outbreak management. Others parameters are $\beta =0.8333, \gamma =0.3333, a=0.01, {D}_{g}={D}_{r}=0.5, h=1.0$ and $e=1.0$.

The introduction of immunity waning (with a parameter $p=0.01$) has a significant impact on the dynamics of the social dilemma structure within the Vaccination Game (VG) (as shown in Fig. 2), as compared to the scenario when $p=0.0$. Specifically, when the relative vaccination cost is low $({C}_{r}=0.1)$, we observe that there is a shorter duration of the PD phase compared to the corresponding case in Fig. 1 (when $p=0.0$). This can be explained by the fact that immunity waning increases an individual's susceptibility to infection. Thus, when the cost of vaccination is low, people are more inclined to rely on vaccination to avoid the increased risk of infection.

However, when the vaccination cost is high (${C}_{r}=0.9$), people become hesitant to rely on vaccination because the cost is comparable to the perceived benefits. Interestingly, this hesitancy leads to fluctuations in people's commitment to vaccination, represented as $V(t)$, which, in turn, results in multiple time intervals of PD.

When examining these time series data (Figs. 1 and 2), we can derive several key insights:

1. *Initiation of the episode* Each episode typically begins in the Trivial game state. This initial state arises because of low infection rates and low vaccination rates (as indicated in Table 1). In such circumstances, individuals tend to comply with social distancing measures.
2. *Rapid transition to PD* This initial Trivial game state rapidly transitions into a social dilemma characterized by PD. This shift occurs as an outbreak starts, creating a situation where individuals face increased infection risks.
3. *Decrease in dilemma intensity* Following the peak of the outbreak, there was a decline in the intensity of the social dilemma. In some cases, the situation may revert to the Trivial game state, but in others, PD continues.
4. *Potential for multiple PD phases* In scenarios with factors such as low vaccine efficacy or high vaccination costs, PD may persist even after the initial outbreak peak (in what we can call the "1st PD region"). This persistence is due to the ongoing social dynamics surrounding individual compliance with social distancing, which remains a crucial issue.

In summary, the introduction of immunity waning influences the dynamics of the social dilemma within the Vaccination Game. The interplay between vaccination costs, immunity waning, and individual behaviors leads to complex and evolving patterns of social distancing and vaccination compliance, reflecting the ongoing challenges in managing disease outbreaks.

### Behavioral steady-state outcomes

#### Cost and effectiveness analysis along “$a$”

**Figure 3:**
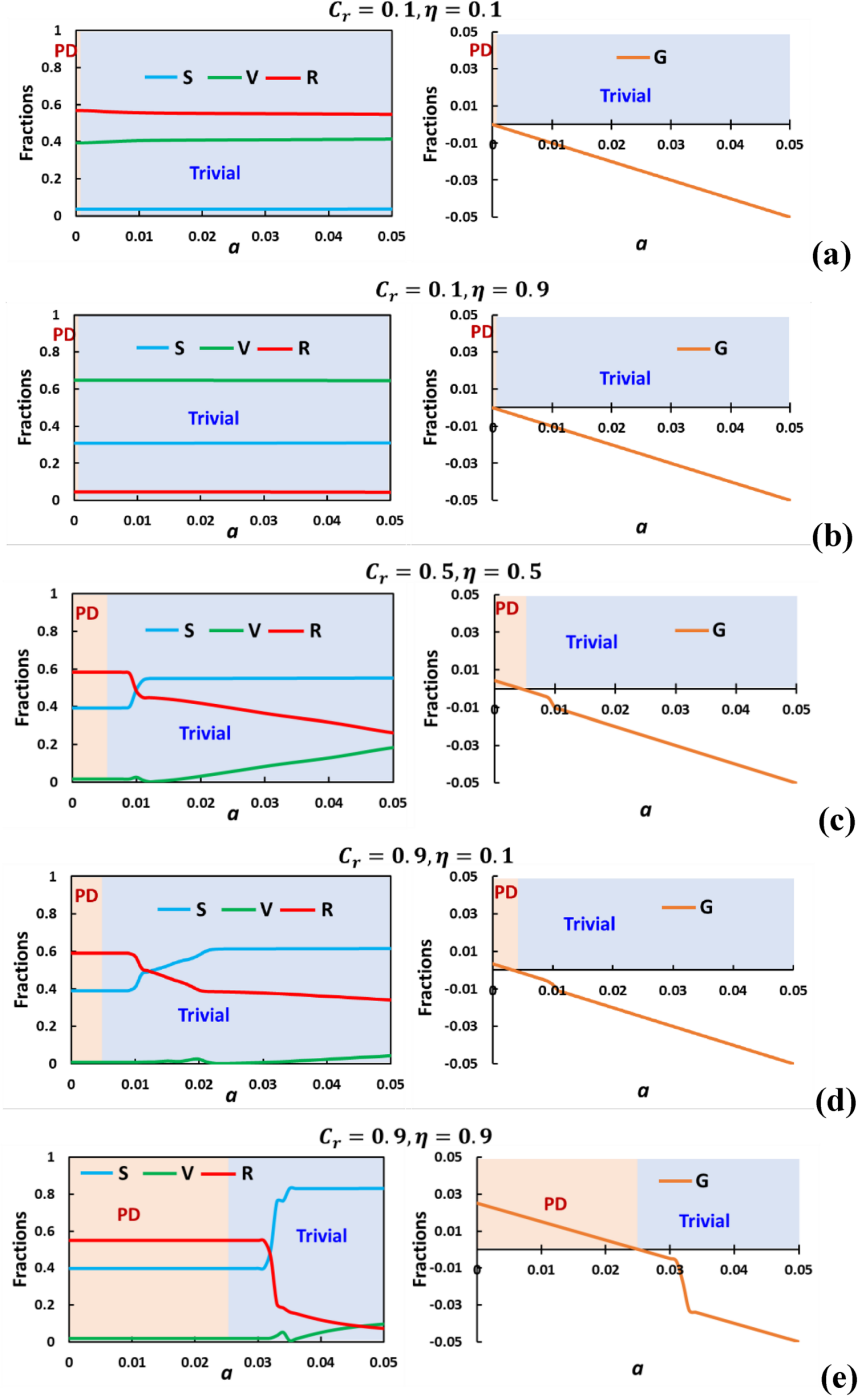
Illustrates a series of line graphs along "$a$" representing various scenarios related to vaccination cost and efficacy, focusing on the final epidemic size (R) colored red, the number of vaccinated individuals (V) colored green, the number of susceptible individuals (S) colored blue, and a parameter denoted as "$G$" colored orange at a steady-state situation as time approaches infinity. The panels labelled (**a**) through (**e**) correspond to different combinations of vaccination cost (${C}_{r}$) and efficacy ($\eta$): (0.1, 0.1), (0.1, 0.9), (0.5, 0.5), (0.9, 0.1), and (0.9, 0.9), respectively. When vaccination cost (${C}_{r}$) is low, creating a "trivial game class", there's no dilemma, and $SED=0$. Regardless of vaccination efficacy $(\eta )$, $G$ remains similar, indicating a trivial situation with higher $\eta$ leading to lower FES due to increased vaccinations. Higher ${C}_{r}$ expands Prisoner's Dilemma (PD) regions, discouraging vaccination. Maximum PD occurs at high ${C}_{r}$ and $\eta$ due to higher reliability. SED is frequent in PD scenarios, with $G$ reflecting dilemmas. In PD regions, fewer adhere to social distancing, while negative $G$ implies more distancing in trivial areas. $G$ serves as a dilemma indicator affecting compliance. Others parameters are $\beta =0.8333, \gamma =0.3333, {D}_{g}={D}_{r}=0.5, h=1.0$ and $e=1.0$.

Figure 3 illustrates a series of line graphs along “$a$” representing various scenarios related to vaccination cost and efficacy, focusing on the final epidemic size (R), the number of vaccinated individuals (V), the number of susceptible individuals (S), and a parameter denoted as "G" at a steady-state situation as time approaches infinity. The panels labeled (a) through (e) correspond to different combinations of vaccination cost (${C}_{r}$) and efficacy ($\eta$): (0.1, 0.1), (0.1, 0.9), (0.5, 0.5), (0.9, 0.1), and (0.9, 0.9), respectively.

When the relative vaccination cost $({C}_{r})$ is low (indicating a lower cost for vaccination), the equilibrium points result in a situation referred to as a "trivial game class". In this scenario, there is no dilemma situation, and the social efficiency deficit (SED) is equal to 0. Comparing panels (a) and (b), we observe that varying the $\eta$ values (vaccination efficacy) does not significantly impact the parameter $G$ along the "$a$" axis. Regardless of whether $\eta$ is low or high, the parameter $G$ exhibits a similar trend, indicating an almost trivial situation. The primary difference is that higher eta values result in lower FES because they lead to a higher number of vaccinated individuals.

However, when the vaccination cost increases, it enlarges the region of Prisoner's Dilemma (PD) situations. Dilemmas emerge due to the higher costs, as seen in panels (c) and (d). At relatively low values of "$a$", the PD region dominates, while increasing "$a$" values leads to more trivial cases. The maximum PD region is observed when both the cost $({C}_{r})$ and vaccine efficacy ($\eta$) are high, specifically for the case when ${C}_{r}=0.9$ and $\eta =0.9$. This occurs because higher $\eta$ values imply greater vaccine reliability. Nevertheless, higher vaccine costs can discourage people from participating in the vaccination program, resulting in a dilemma situation. SED is most frequently observed when dealing with the PD game class, and the new indicator $G$ somehow reflects the dilemma situation.

In terms of the $G$ values, it's observed that when the PD region prevails, fewer susceptible individuals adhere to social distancing measures. In contrast, in the trivial region, where negative $G$ values are prominent, people tend to engage in more social distancing behavior. This suggests that $G$ can serve as an indicator of the level of dilemma within the population and its impact on individuals' compliance with social distancing measures.

#### Parameters "$e$" and "$h$" analysis along “$a$”

**Figure 4:**
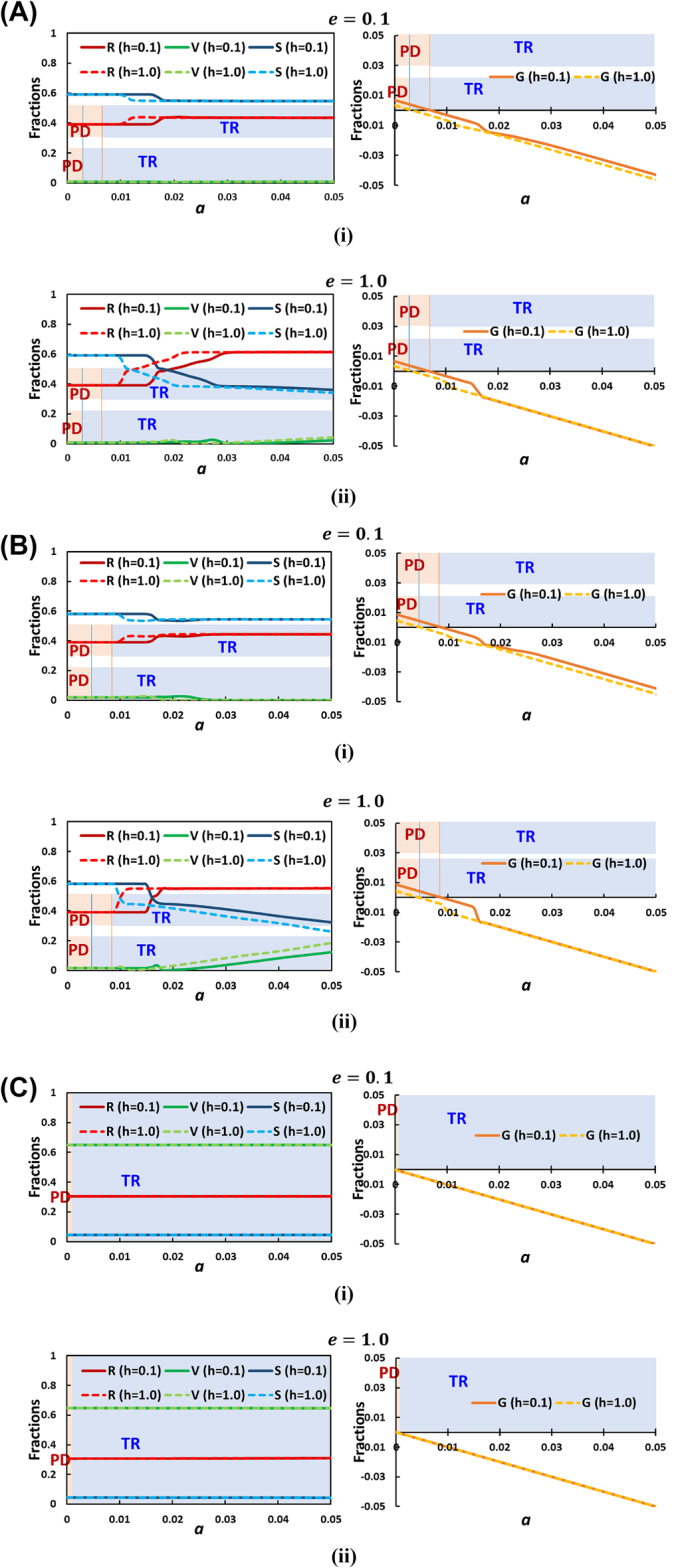
(**A**) Detailed Analysis of Parameters "$e$" and "$h$" with Respect to "$a$". This figure presents a comprehensive analysis of the parameters "$e$" and "$h$" in the context of (${C}_{r}=0.9,\eta =0.1$) in which Two sub-panels labelled (i) and (ii) correspond to cases with "$e$" equal to 0.1 and 1.0, respectively. (**A**) Depicts two panels for "$e$" values of 0.1 and 1.0, respectively, with varying "$h$" values. Interestingly, "$e$" variations (0.1 or 1.0) show no significant impact on the observed trend. Whether "$e$" is low or high, its variation does not notably affect the outcome. Others parameters are $\beta =0.8333, \gamma =0.3333, \text{and} {D}_{g}={D}_{r}=0.5$. (**B**) Detailed analysis of parameters "$e$" and "$h$" with respect to "$a$". This figure presents a comprehensive analysis of the parameters "$e$" and "$h$" in the context of (${C}_{r}=0.5,\eta =0.5$) in which Two sub-panels labelled (i) and (ii) correspond to cases with "$e$" equal to 0.1 and 1.0, respectively. Lowering "$h$" ($h=0.1$) once more expands the Prisoner's Dilemma (PD) region, while "$e$" variation does not notably impact the outcome. The observed behavior remains largely unchanged whether "$e$" is set to 0.1 or 1.0. This suggests that "$e$" lacks significant sensitivity to variations, consistently influencing the observed dynamics across different scenarios. Others parameters are $\beta =0.8333, \gamma =0.3333, \text{and} {D}_{g}={D}_{r}=0.5$. (**C**) Detailed analysis of parameters "$e$" and "$h$" with respect to "$a$". This figure presents a comprehensive analysis of the parameters "$e$" and "$h$" in the context of (${C}_{r}=0.1,\eta =0.9$) in which Two sub-panels labelled (i) and (ii) correspond to cases with "$e$" equal to 0.1 and 1.0, respectively. When vaccine cost is low and efficacy high, "$e$" and "$h$" variations have minimal impact on outcomes. While lower "$h$" values promote the Prisoner's Dilemma (PD) scenario, "$e$" lacks notable influence. Overall, "$h$" affects the transition from PD to Trivial stage, whereas "$e$" appears insignificant. Others parameters are $\beta =0.8333, \gamma =0.3333, \text{and} {D}_{g}={D}_{r}=0.5$.

Let's delve into a detailed analysis of two crucial parameters, "$e$" and "$h$". Figure 4A–C provide a series of line graphs with respect to the parameter "$a$". These graphs correspond to different combinations of vaccination cost (${C}_{r}$) and efficacy ($\eta$): $({C}_{r}=0.9, \eta =0.1), ({C}_{r}=0.5, \eta =0.5),$ and $({C}_{r}=0.1, \eta =0.9)$, respectively. Each figure contains two panels labeled (i) and (ii), representing cases with "$e$" equal to 0.1 and 1.0. Let's start by examining Fig. 4A, where the lower value of "$h$" ($h=0.1$) appears to enlarge the region characterized by the Prisoner's Dilemma (PD) compared to the case with a higher "$h$" value ($h=1.0$). In this situation, we observed that the parameter "$e$" doesn't seem to have a significant impact. Whether "$e$" is set to 0.1 or 1.0, there is no noticeable sensitivity to its variation. Moving on to Fig. 4B, which corresponds to $({C}_{r}=0.5, \eta =0.5)$, we observe a similar trend. The lower "$h$" value ($h=0.1$) again extends the PD region, and once more, "$e$" does not exhibit any discernible sensitivity. Whether "$e$" takes on a value of 0.1 or 1.0, the behavior remains largely unchanged.

Finally, in Fig. 4C, we examine the scenario where the relative vaccine cost is low and efficacy is high (${C}_{r}=0.1, \eta =0.9$). Across different values of "$e$" and "$h$", no significant sensitivity is observed. This suggests that when the vaccine cost is low and its efficacy is high, variations in "$e$" and "$h$" do not seem to have a pronounced impact on the outcome. Thus, after considering all three cases, we can conclude that while "$h$" does have some influence on transitioning from the PD stage to the Trivial stage, "$e$" appears to have little to no significant effect. The lower "$h$" values tend to promote the PD scenario, but "$e$" doesn't seem to play a pivotal role in altering the observed tendencies.

### Phase plane analysis

#### $"{\beta }_{0}"$ versus $"a"$ phase plane

**Figure 5:**
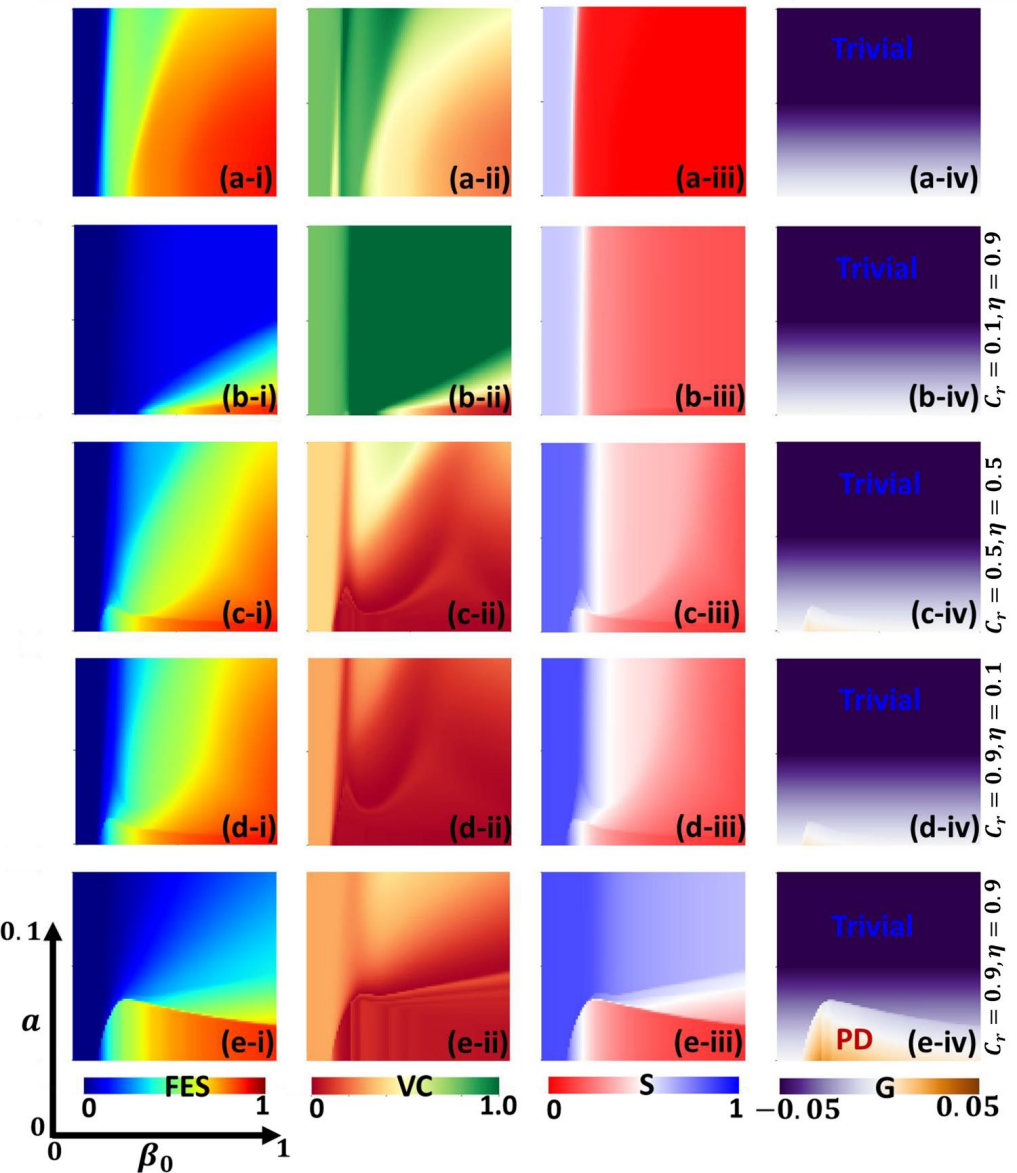
Phase diagram of equilibrium points. We illustrate a range of results based on two pivotal parameters: the constant disease treatment rate $({\beta }_{0})$ and "game compassion", denoted as "$a$." These parameters characterize the underlying social dilemmas associated with vaccination and social distancing strategies. Here, Panels (*-i) Depict the final epidemic size (FES), Panels (*-ii) Display vaccination coverage (VC), Panels (*-iii) Show the number of susceptible individuals (S), and Panels (*-iv) Present an indicator denoted as "$G$". Meanwhile, the sub-panels labelled (a-*), (b-*), (c-*), (d-*), and (e-*) represent the outcomes of varying vaccine cost and efficiency for the following combinations: (0.1, 0.1), (0.1, 0.9), (0.5, 0.5), (0.9, 0.1), and (0.9, 0.9), respectively. In the FES graph, the blue region represents disease-free equilibrium (DFE), notably observed when ${\beta }_{0}<\gamma$. Elevated transmission rates and lower game compassion (a) correlate with higher final epidemic size (FES). Conversely, higher values lead to reduced FES due to the expanded Trivial (TR) region, where social distancing is common, slowing transmission. Panels (a-*) and (b-*) show vaccination impacts: moderate uptake reduces FES, while high vaccine reliability drastically lowers FES, enhancing DFE. Panels (c-*), (d-*), and (e-*) reveal decreasing interest in vaccination with rising costs. Panel (e-*) stands out, showing a societal dilemma with high costs and reliability, expanding the Prisoner's Dilemma (PD) region, and increasing FES. In the trivial region, increased susceptibility prompts more social distancing, controlling disease spread effectively. Others parameters are $\gamma =0.1, {D}_{g}={D}_{r}=0.5, h=1.0$ and $e=1.0$.

In Fig. 5, alongside the conventional line graphs, we introduce a new set of results represented as a phase diagram. This phase diagram depicts the equilibrium point and is characterized by two key parameters: the constant disease treatment rate ($\beta$) and game compassion, $a$, which describe the fundamental social dilemmas associated with vaccination and social distancing strategies. Panels (*-i), (*-ii), (*-iii), and (*-iv) display the final epidemic size ($FES$), vaccination coverage ($V$), susceptible individuals ($S$), and the indicator, $G$. Meanwhile, panels (a-*), (b-*), (c-*), (d-*) and (e-*) show the result of varying the vaccine cost and efficiency: (0.1, 0.1), (0.1, 0.9), (0.5, 0.5), (0.9, 0.1), and (0.9, 0.9), respectively. The blue region within the *FES* graph signifies the disease-free equilibrium (DFE) that was observed thoroughly in Panels (*-i), t. Notably, the left DFE region is observed when ${\beta }_{0}<\gamma$. It's important to highlight that a higher final epidemic size ($FES$) is observed when disease transmission rates are elevated and "$a$" (game compassion) is lower. Conversely, a higher value of "$a$" results in a reduced FES due to the expansion of the Trivial (TR) region, as indicated in panel (*-iv). In the TR region, individuals tend to engage in social distancing practices. This increased social distancing behavior slows down disease transmission, affording people more time to contemplate participating in vaccination programs.

In Panel (a-*), regardless of lower vaccine efficacy, a moderate number of individuals opt for vaccination, leading to a reduction in $FES$. In contrast, in Panel (b-*), where the vaccine is highly reliable ($\eta =0.9$), more people participate in vaccination programs, resulting in a substantial reduction in FES and an enhancement of the disease-free equilibrium. Interestingly, in both scenarios, the presence of susceptible individuals ($S$) is observed only within the DFE region when ${\beta }_{0}<\gamma$.

Moving on to Panels (c-*), (d-*), and (e-*), these panels reveal a notable trend where individuals display reduced interest in participating in vaccine programs as vaccine costs increase. However, Panel (e-*) stands out as particularly intriguing. In this Panel, the vaccination cost is set at a high value $({C}_{r}=0.9)$, and the vaccine remains remarkably reliable $(\eta =0.9)$. In this scenario, where there's a juxtaposition of elevated costs and high reliability, a societal dilemma emerges, leading to the expansion of what we'll refer to as the PD region. This PD region, as depicted in Panel (e-i), corresponds to a higher final epidemic size (FES), as mirrored in the G graphs of Panel (e-iv). It's important to draw attention to the findings within the trivial region shown in Panel (e-iv), where we observe an increase in the number of susceptible (S) individuals, as illustrated in Panel (e-iii). This surge in susceptible individuals contributes to the reduction of FES. In this situation, people predominantly opt for social distancing measures in their efforts to protect themselves from disease transmission, effectively controlling and diminishing the rate of disease spread.

#### $"\eta "$ versus $"{C}_{r}"$ phase plane

**Figure 6:**
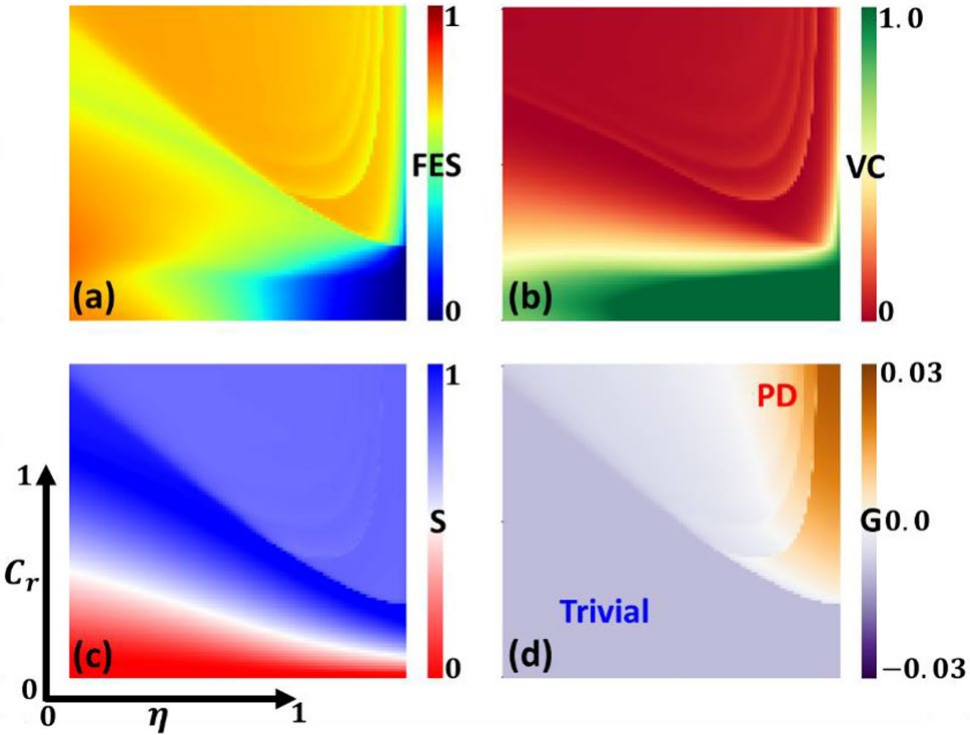
We present a set of 2D phase diagrams featuring panels (**a**), (**b**), (**c**), and (**d**), representing various aspects, including the final epidemic size (FES), vaccine coverage (VC), susceptible individuals (S), and the parameter $G$, respectively. These aspects are examined in relation to vaccination efficacy ($\eta$) and vaccination cost (${C}_{r}$). High vaccine efficacy and low cost foster widespread vaccination uptake, reflecting the Nash Equilibrium. Cooperation prevails, minimizing dilemmas, notably in trivial scenarios. However, endemic equilibrium emerges in conditions of lower vaccine reliability and higher cost, manifesting in increased reliance on social distancing. This shift occurs when vaccination affordability and efficacy pose barriers, prompting individuals to resort to distancing measures instead of vaccination. Others parameters are $\beta =0.8333, \gamma =0.3333, a=0.01, {D}_{g}={D}_{r}=0.5, h=1.0$ and $e=1.0$.

Recent research on vaccination games has shed light on the emergence of vaccine-related attitudes and dilemmas, which are influenced by factors such as vaccine cost and efficiency. However, people's attitudes can vary significantly when it comes to the balance between incentives and disincentives. The current study delves into the impact of self-regarding preferences on individuals' decisions to participate in voluntary vaccination efforts and their efforts to protect themselves from contagious diseases. To achieve this objective, we present a 2D phase diagram in Fig. 6, featuring panels (a), (b), (c), and (d), representing various aspects, including the final epidemic size (FES), vaccine coverage (VC), susceptible individuals (S), and the parameter G, respectively. These aspects are examined in relation to vaccination cost and vaccination efficacy. It's crucial to acknowledge that when vaccination is highly reliable, and its cost is low, it results in nearly universal vaccine coverage. This means that virtually all individuals choose to participate in the vaccination program as a proactive measure to safeguard themselves against the disease. This implies that cooperation represents the optimal solution in this context, aligning with the Nash Equilibrium (NE). As anticipated, the region marked by purple (negative) appears in parameter G. This absence of dilemmas is particularly evident in the trivial game. However, an endemic equilibrium is observed in the final epidemic size (FES) in Panel (a) when vaccine reliability is lower and vaccination cost is higher. In such circumstances, people tend to gravitate towards social distancing measures, especially when the cost of vaccination is high, and its efficacy is low. This outcome is quite plausible, as people are more inclined to adopt social distancing strategies when vaccination is not a feasible option due to either cost constraints or doubts about its efficiency.

The primary message conveyed in this manuscript is the ability to quantify the evolving nature of social dilemmas through the parameter $G$. Until now, the existing Social Efficiency Deficit ($SED$) framework^28^ allowed for the assessment of an overall index representing the extent of dilemmas (not 'dilemma strength,' which should be emphasized) but lacked a specific quantitative index. In this context, $G$ can be seen as an alternative index to SED, serving as a quantitative measure for the extent of social dilemmas. A noticeable distinction exists between $SED$ and $G$. $SED$ ranges from 0 to a theoretically infinite positive value, with $SED=0$ indicating consistency between the Nash equilibrium and the Social Optimal (SO) state. Conversely, a positive $SED$ implies a deviation from SO, indicating the degree of the social dilemma. In relation to ${D}_{g} \text{and} {D}_{r}$, a positive (negative) value of $G$ signifies the presence of a Prisoner's Dilemma or Trivial scenario.

In conventional Vaccination Games (VGs), as developed by Fu^31,32^ and followed by many others^3,13,17^, an aspect of evolutionary game theory was not explicitly incorporated. Such VG models combine epidemic dynamics (using SIR models, for instance) and agent decision dynamics, where individuals decide whether to vaccinate based on expected payoff comparisons with and without vaccination. Consequently, these models inherently introduce a social dilemma structure. Our study, utilizing the concept of SED, revealed that comparing expected payoffs at Nash equilibrium, resulting from episodes based on assumed VGs, to those obtained from the Social Optimal case effectively quantifies the deviation from a Trivial game, free of dilemmas. Therefore, a positive $SED$ inherently suggests the presence of a social dilemma, while $SED=0$ indicates a dilemma-free situation. However, it's important to note that $SED$ can only assess the overall dilemma extent over the entire episode's time evolution and does not pinpoint whether a social dilemma exists at a specific moment within an episode, which typically comprises initial epidemic growth, peak infection, decline, and disease eradication.

In contrast, our proposed framework in this study defines the instantaneous extent of dilemmas (dilemma strength) by introducing $G(t)$. In fact, some of the time series data presented in Figs. 1 and 2 clearly demonstrate that a Prisoner's Dilemma-type social dilemma arises when vaccination levels are insufficient to control the epidemic, and this can occur multiple times within a single episode. Therefore, our presented model allows for the evaluation of the evolving dynamics of whether a social dilemma is active and to what extent it operates explicitly. This novel approach illuminates the dual dynamics of epidemics and human behavior, revealing whether a specific social dilemma exists at a given moment or not.

## Conclusion

A critical aspect to consider is that individuals who receive vaccinations confer certain benefits to both them and those around them, whether these benefits arise from cooperative or defective behaviors. However, it is essential to acknowledge that some individuals may harbor concerns about the cost, efficiency, or potential harm associated with participating in vaccination programs. In each case, individuals' decisions are influenced by their past actions and memories of previous outcomes, which in turn contribute to the collective welfare of the group. The coupled epidemiological-evolutionary Game Theory (EGT) model presented here addresses this nuanced aspect. It combines epidemic dynamics modeled using the Susceptible-Vaccinated-Infected-Recovered (SVIR) framework with pairwise game dynamics to quantify the impact of social distancing explicitly. The most important advantage of the present model of vaccination game (VG) vis-a-vis the previous VG models is that the time-evolving social dilemma structure, whether non-dilemma (Trivial) or dilemma (Prisoner’s Dilemma) works behind at every time-phase, i.e., at the very initial moment after initial infectious individuals appearing, a transient time from the initial moment to rapid growth period, peak time, and disease cool-down period, can be explicitly predicted.

Through extensive numerical simulations, this study successfully highlights the central theme discussed above. The model's versatility is underscored as it explores vaccination behavior and its reliability in conjunction with cooperative behaviors that encompass various manifestations of the Prisoner's Dilemma (PD) and Trivial game scenarios. The introduced index, $G$, is a novel quantifier in this context, shedding light on the relationship between Disease Spread (DS), Social Equilibrium Dynamics (SED) parameters, and the extent of dilemmas. Therefore, this study presents the potential to examine the interplay between SED, G, and the strength of social dilemmas as a more comprehensive index for elucidating their presence. The research underscores the emphasis on quantifying the evolving nature of social dilemmas through $G(t)$, positioning it as a valuable alternative to the existing SED framework. While SED provides a broad measure of dilemma extent, $G(t)$ offers a direct and quantitative assessment of dilemma strength, particularly distinguishing scenarios such as the Prisoner's Dilemma (PD) and Trivial games.

In conventional Vaccination Games (VGs), the incorporation of evolutionary game theory was previously lacking, limiting our understanding. By utilizing SED, this study identifies social dilemmas by contrasting expected payoffs at the Nash equilibrium with those in the Social Optimal case, thereby quantifying deviations from a dilemma-free state. Notably, the introduction of G(t) enables the assessment of instantaneous dilemma strength, unveiling moments when social dilemmas arise during various stages of epidemic episodes. Consequently, this novel approach sheds light on the intricate interplay between disease dynamics and human behavior. In conclusion, this research introduces $G(t)$ as a potent tool for dissecting the dynamics of social dilemmas, offering fresh insights into their temporal evolution, and enhancing our understanding of the complex relationship between individual decisions and disease dynamics. Therefore, the study's analysis reveals complex dynamics in vaccination decision-making, offering insights into policy and healthcare strategies. Recognizing individual concerns about vaccination costs and safety can guide targeted communication efforts. Understanding evolving social dilemmas throughout epidemics enables tailored interventions to encourage vaccination while minimizing their impact. The introduced index, $G(t)$, allows policymakers to assess vaccination campaign effectiveness over time and design strategies promoting cooperative behaviors. Overall, the study underscores the importance of nuanced approaches to vaccination programs for improved public health outcomes.

## Data Availability

The datasets generated and/or analyzed during the current study are not publicly available due to original code but are available from the corresponding author on reasonable request.
